# MicroRNAs from Extracellular Vesicles Secreted by Bovine Embryos as Early Biomarkers of Developmental Competence

**DOI:** 10.3390/ijms21238888

**Published:** 2020-11-24

**Authors:** Bárbara Melo-Baez, Yat S. Wong, Constanza J. Aguilera, Joel Cabezas, Ana C. F. Mançanares, Gonzalo Riadi, Fidel O. Castro, Lleretny Rodriguez-Alvarez

**Affiliations:** 1 Laboratory of Animal Biotechnology, Department of Animal Science, Faculty of Veterinary Sciences, Universidad de Concepción, Av. Vicente Mendez 595, Chillan 3780000, Chile; barbaramelo@udec.cl (B.M.-B.); ywong@udec.cl (Y.S.W.); consaguilera@udec.cl (C.J.A.); joelcabezas@gmail.com (J.C.); anamancanares@gmail.com (A.C.F.M.); fidcastro@udec.cl (F.O.C.); 2 ANID-Millennium Science Initiative Program Millennium Nucleus of Ion Channels-Associated Diseases (MiNICAD), Center for Bioinformatics, Simulation and Modeling, CBSM, Department of Bioinformatics, Faculty of Engineering, Campus Talca, University of Talca, Talca 3460000, Chile; griadi@utalca.cl

**Keywords:** IVF, bovine, embryo, microRNAs, biomarkers

## Abstract

During early development, embryos secrete extracellular vesicles (EVs) that participate in embryo–maternal communication. Among other molecules, EVs carry microRNAs (miRNAs) that interfere with gene expression in target cells; miRNAs participate in embryo–maternal communication. Embryo selection based on secreted miRNAs may have an impact on bovine breeding programs. This research aimed to evaluate the size, concentration, and miRNA content of EVs secreted by bovine embryos with different developmental potential, during the compaction period (days 3.5–5). Individual culture media from in vitro–produced embryos were collected at day 5, while embryos were further cultured and classified at day 7, as G1 (conditioned-culture media by embryos arrested in the 8–16-cells stage) and G2 (conditioned-culture media by embryos that reached blastocyst stages at day 7). Collected nanoparticles from embryo conditioned culture media were cataloged as EVs by their morphology and the presence of classical molecular markers. Size and concentration of EVs from G1 were higher than EVs secreted by G2. We identified 95 miRNAs; bta-miR-103, bta-miR-502a, bta-miR-100, and bta-miR-1 were upregulated in G1, whereas bta-miR-92a, bta-miR-140, bta-miR-2285a, and bta-miR-222 were downregulated. The most significant upregulated pathways were fatty acid biosynthesis and metabolism, lysine degradation, gap junction, and signaling pathways regulating pluripotency of stem cells. The characteristics of EVs secreted by bovine embryos during the compaction period vary according to embryo competence. Embryos that reach the blastocyst stage secrete fewer and smaller vesicles. Furthermore, the loading of specific miRNAs into the EVs depends on embryo developmental competence.

## 1. Introduction

During the last years, molecular transfer within extracellular vesicles (EVs) has been described as a new mechanism for cellular communication [1,2]. These are released to the extracellular environment by different cell types and are heterogeneous in origin, size, and content [3]. Due to their content (mRNAs, small RNAs, proteins, lipids, and DNA) and their ability to transfer signaling molecules to receptor cells, EVs have been proposed as disease biomarkers and potential therapeutic agents [4,5].

EVs are involved in several physiologic process, including gamete development [6] and embryo–maternal interaction, even before the onset of molecular signaling of pregnancy recognition [7]. Preimplantation embryos from different species, including human, pig, mouse, and bovine, secrete EVs that can modify gene expression in maternal cells or in other embryos [8,9,10]. The characteristics of EVs, including their concentration, size, distribution, and content, might reflect embryo quality, thus making them potential markers of embryonic developmental competence [10,11,12,13]. In fact, the most competent bovine embryos secrete fewer but larger EVs during blastulation [12,13].

Embryos secrete microRNAs (miRNAs) involved in physiological phenomena, such as the proliferation and growth of endometrial cells, making EVs a potential communication system between the developing embryo and the endometrium [14]. Recently, Mellisho et al. [12] found miRNAs differentially regulated in EVs released during blastulation by bovine embryos with different developmental competence. In that work, miR-2284ab was the most upregulated miRNA in EVs from more competent embryos. Song et al. [15] found miR-2284ab upregulated during the receptive phase of goat’s endometrium. Good-quality preimplantation embryos seem to secrete EVs transporting miRNAs that improve endometrium receptivity. However, Mellisho et al. [12] also proposed that some upregulated miRNAs (bta-miR-1 and bta-miR-184) in EVs from good-quality embryos block cell functions that are required for normal embryo development. This might suggest another biological role of EVs as a mechanism for disposal of molecules that might be detrimental for embryonic development [12].

At present, the study of EVs cargo has been limited to the period of blastulation, when first cell differentiation takes place. This is a crucial period where the establishment of two different cell types is commanded by differential gene expression [16]. Considering that during the biogenesis of EVs, especially of exosomes, molecules that reflect the functionality of the cell are packed, it is logical to think that changes in gene expression would modify the content of these vesicles. This hypothesis is in concordance with the differential RNA cargo (mRNA and miRNA) found in EVs from embryos with different developmental competence [8,12] and differential embryonic gene expression [8,12,17]. We hypothesize that the secretion of EVs, their morphological characteristics, and cargo will vary according to the critical periods of preimplantation development where major changes in gene expression take place, i.e., at embryonic genome activation (EGA) and during blastulation. In bovine embryos, EGA occurs at the eight-cell stage, and it is characterized by an active transcription of embryo genome that occurs during the last phase of the maternal-to-embryonic transition (MET). This involves the depletion of maternal mRNA, the replacement of maternal transcripts, and the expression of new embryonic transcripts [18]. The degradation of maternal transcripts is driven by several mechanisms, including miRNAs that are expressed by the embryo genome during the minor embryonic transcription stage [19,20]. Based on that, we postulate that changes in the content of miRNAs in embryonic cells during EGA will be reflected in the content of secreted EVs, which in turn will vary in an embryo competence-dependent manner. Therefore, in this work, we focus on the study of the characteristics of EVs secreted during EGA, including their concentration, size distribution, and miRNA cargo, and how these features are influenced by embryo competence and their ability to reach blastocyst stage.

## 2. Results

Bovine embryos were produced by in vitro fertilization (IVF); 1228 zygotes were cultured in groups in synthetic oviduct fluid (SOF) medium. At day 3.5 after IVF (day 0 = day of IVF), embryos at 8–16 cells and with good morphological quality (*n* = 552) were collected and placed in individual culture in EVs-depleted SOF medium (SOFd). At day 5, embryo development was evaluated, and culture medium from each embryo was collected and kept for further analysis. Embryos that did not reach the morulae stage were classified as blocked, and their corresponding culture medium (conditioned medium) was classified as group 1 (G1; *n* = 250). Morulae were kept in individual culture up to day 7, when embryo development was evaluated. Overall, 51% developed to blastocyst stage, and their corresponding culture medium (from day 3.5 to 5) was collected at day 5; they were classified as group 2 (G2; *n* = 154).

### 2.1. Identification and Characterization of EVs from Conditioned-Culture Media by Early Embryos

**Figure 1:**
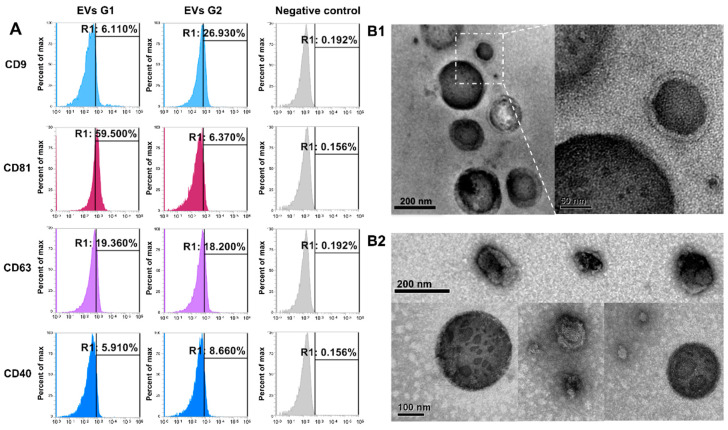
Characterization of extracellular vesicles (EVs) secreted by early bovine embryos with different developmental potential. (**A**) Flow cytometry analysis of EVs markers (CD9, CD63, CD81, and CD40) in EVs secreted by embryos arrested in the 8–16-cells stage (G1) and EVs secreted by embryos that reached blastocyst stages at day 7 (G2). Negative control: beads without EVs and incubated with antibodies against each marker. (**B**) Representative images from transmission electron microscopy (TEM) showing EVs in medium secreted by embryos arrested in the 8–16-cells stage (**B1**) and by embryos that reached blastocyst stages at day 7 (**B2**).

Nanoparticles separated from embryo-conditioned culture media were subjected to flow cell cytometry, the results of which allowed us to classify them as EVs, by the expression of the specific surface markers CD9, CD81, CD63, and CD40 in both experimental groups, but not in the negative control (Figure 1A). Further EVs were visualized by transmission electron microscopy (TEM). Classical membrane-enclosed particles resembling EVs were identified in each experimental group (Figure 1(B1,B2)) and classified as EVs by the identification of specific surface markers CD9, CD81, CD63, and CD40 (Figure 1A). All of those markers were identified in EVs from both experimental groups (G1, EVs from embryos arrested in the 8–16-cells stage; G2, EVs from embryos that reached blastocyst stages at day 7) but not in the negative control.

**Figure 2:**
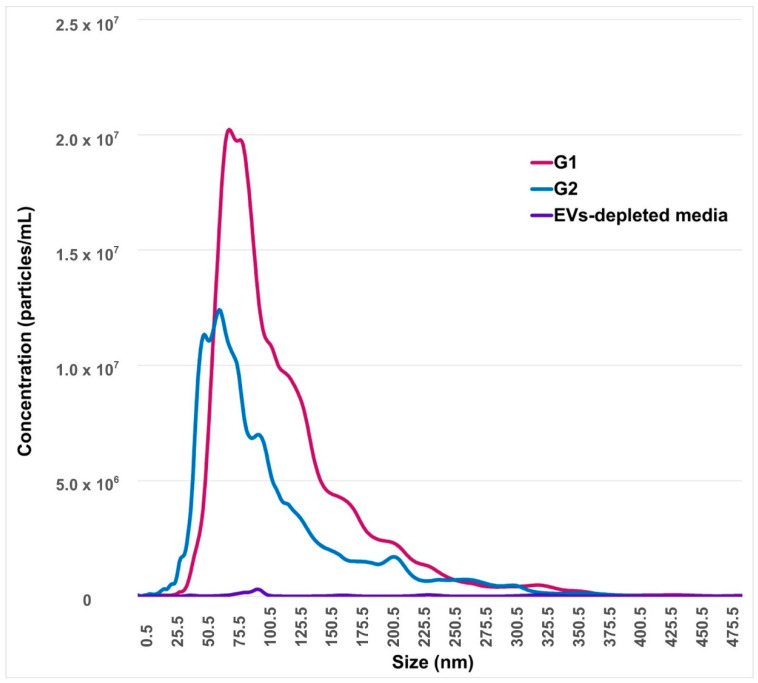
Nanoparticles tracking analysis of embryo-conditioned culture media. The horizontal bars indicate particle size range, while the y-axis indicates concentration of nanoparticles according to EVs size. G1: conditioned-culture media by embryos arrested in the 8–16-cells stage. G2: conditioned-culture media by embryos that reached blastocyst stages at day 7. EVs-depleted synthetic oviduct fluid (SOF) medium is considered as the negative control.

The concentration of EVs secreted by embryos that reached blastocyst stages at day 7 (G2) was 9.4 × 10^8^ particles/mL, while the mean and mode of size were 109.4 and 89.1 nm, respectively. All of these values were significantly lower (*p* < 0.05) compared to arrested embryos at the 8–16-cells stage (G1), where the concentration of EVs was 1.5 × 10^9^ particles/mL, and the mean and mode of size were 129.6 nm and 108.3 nm, respectively (Figure 2).

### 2.2. miRNAs Differential Expression Analysis

**Figure 3:**
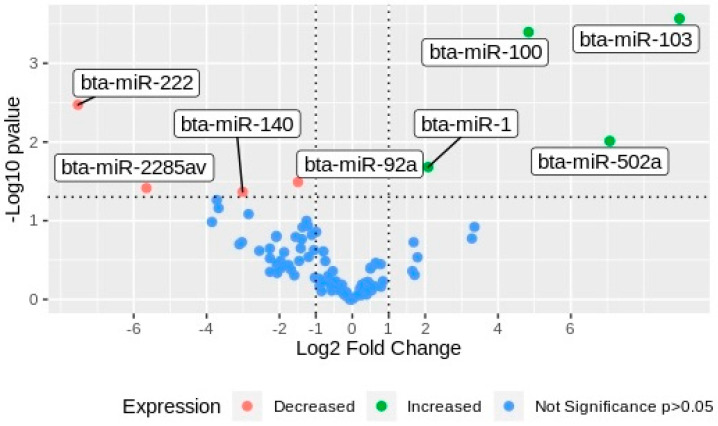
Volcano plot of miRNAs differentially expressed in EVs from embryos with different development potential. miRNAs were plotted according to the values of Log2 fold change in the horizontal bar against the Log10 of the *p*-values in the y-axis. Green dots, miRNAs upregulated in EVs secreted by arrested embryos (G1); red dots, miRNAs downregulated in G1; blue dots, miRNAs non-differentially expressed between groups.

After NTA, 3 pools of media coming from 10 individual cultured embryos per group were organized for next-generation sequencing (NGS) of small RNA. All RNAs from each biological replicate were isolated and quantified before library construction and NGS. Using SRNA bench pipeline, the adapters were removed, and the reads were filtered according to their quality and length, resulting in 24.336.044 reads that were useful for miRNA mapping. The reads were mapped against the bovine reference genome (ARS-UCD 1.2) in miRbase. Only the gene counts with CPM ≥ 5 were selected for differential expression analysis. A total of 95 miRNAs were expressed across the samples (Table 1), and eight miRNAs were differentially expressed between experimental groups (G1 vs. G2). Four miRNAs, namely bta-miR-103, bta-miR-100, bta-miR-502a, and bta-miR-1, were upregulated in EVs secreted by arrested embryos (G1), whereas bta-miR-92a, bta-miR-140, bta-miR-2285av, and bta-miR-222 were downregulated (Figure 3).

**Figure 4:**
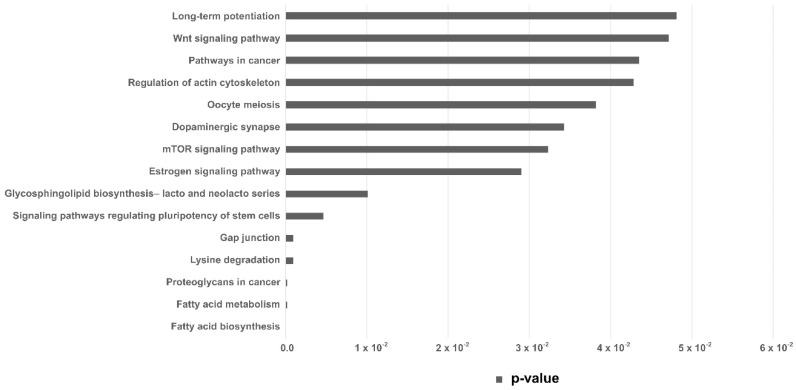
KEGG pathways analysis. KEGG pathways predicted from the miRNAs upregulated in EVs secreted by arrested embryos at 8–16 cells. Horizontal bar indicates *p*-value.

Pathway enrichment analysis was performed with the set of differentially expressed miRNAs in the EVs from embryos arrested in the 8–16-cells stage, revealing 5 KEGG (Kyoto Encyclopedia of Genes and Genome) pathways (*p* < 0.005). Those were predicted from the number of target genes and miRNAs involved (Table 2). KEGG pathways with *p* < 0.05 are shown in Figure 4.

## 3. Discussion

This research was aimed to evaluate the characteristics and miRNA content of EVs secreted by competent and non-competent bovine embryos during cell compaction (after embryonic genome activation (EGA) up to morula). Embryo developmental potential was considered to organize two groups of EVs: Non-competent embryos were those blocked during compaction (G1), while competent embryos were those that developed to blastocyst stage (G2). The nanoparticles collected from each experimental group were characterized as EVs, using the criteria suggested by Théry et al. [21]. According to those criteria, the particles were evaluated by NTA, TEM, and flow cytometry. Nanoparticles collected from embryo culture media were classified as EVs by considering their morphology, which was evaluated by TEM and by the presence of surface markers (CD9, CD81, CD63, and CD40), all of which were absent in the negative control. We found low positivity of surface markers in the EVs analyzed; here we used latex beads binding to EVs prior to antibody incubation, to assess the presence of surface markers. The low amount of EVs collected from preimplantation, embryos and therefore the low number of EVs-beads complexes, might explain the low positivity to surface markers. This pattern has been observed by others in EVs secreted by preimplantation bovine embryos [11,12].

The size of the EVs secreted by competent embryos that developed to blastocysts was around 100 nm, and it is smaller (*p* = 0.0003) than the mean size of the EVs released by arrested embryos. Competent embryos also secrete a lower amount of EVs, compared to blocked embryos. Other studies have reported the size and concentration of EVs in human and bovine embryo and their potential implications for selection of more competent embryos [9,10,11,12,13,22,23,24]. Several reports have shown that both the size and concentration of EVs are lower in competent human embryos at days 3 and 5 after fertilization [22,23,24], which is in concordance with our results. In discordance with our results, Dissayanake et al. [13] reported no significant differences in the concentration or the size of EVs secreted by bovine embryos from day 0 to 5, independent of their quality. This difference may be due to experimental design; in our work, EVs were collected during a narrower window of embryonic development (days 3.5–5). Nevertheless, the same group acknowledged that, at days 7–8, competent and good quality embryos secreted fewer but larger EVs [12,13].

In summary, it is a fact that the characteristics of EVs vary during preimplantation embryo development; there seems to be a shift to lesser secretion of vesicles when embryos are of good quality. This change on the secretion of EVs could be associated with cellular changes that take place during cell compaction and blastulation. The quality of the embryo is associated with the success of these biological events, which could explain the change in the population of EVs according to the competence of the embryo. The reports are still limited, but this work and the current literature suggest that EVs’ characteristics, like size and concentration, may be quantitative parameters that could be of value for embryo classification.

It has been demonstrated that embryo-derived EVs participate in the communication with the maternal side and are internalized by oviductal [25] and the endometrial cells [26]. Molecules loaded into the EVs can alter the function of receptor cells, preparing the maternal side for implantation and further embryo development. However, the content of EVs released by preimplantation embryos also varies according to embryo competence [12,24], which is supposed to affect embryo–maternal crosstalk. Mellisho et al. [12] reported eight miRNAs present only in EVs secreted by competent bovine embryos during the blastulation period. The change in the molecular content of EVs secreted by embryos with different developmental competence may be due to the variations in gene expression that are often observed between these embryos [17]. It would be tempting to hypothesize that if the content of miRNAs in the cytoplasm of the cells varies, the content loaded in the vesicles could also change. However, the question would be, how early are these changes evident in embryonic secretion?

In this study, the content of miRNA within EVs secreted during the compaction window (days 3.5–5) by bovine embryos with different development potential was evaluated. Embryos secrete miRNAs that participate in embryo–maternal communication. The miRNA secretion varies according to embryo kinetic or developmental competence [12,27,28]. The identification of specific miRNAs related to embryo quality may be a non-invasive method that improves the selection of embryos with higher chance of implantation, hence the success of bovine breeding programs. Almost 80% of identified miRNAs were also detected in EVs secreted during bovine embryo blastulation (days 5–7.5) [12]. Moreover, detected miRNAs such as bta-miR-1, bta-miR-10b, and bta-miR-184 have been detected in culture medium of bovine embryos and related to embryo competence [12,27,28].

In this work, eight miRNAs were differentially expressed between G1 and G2. The miRNAs bta-miR-103, bta-miR-100, bta-miR-1, and bta-miR-502a were downregulated in EVs released by competent embryos (G2), and they participate in pathways related to fatty acid biosynthesis and metabolism, lysine degradation, gap junction, and regulating pluripotency of stem cells. Using DIANA-Tarbase v8, we identified 14 genes (FASN, ACADSB, ACSL3, ACOX1, HADH, CPT2, ACADVL, SCD, ACSL4, ACSL1, HSD17B12, ECHS1, and ACAT1) regulated by miR-103 that can modulate the fatty acid metabolism pathway. Recently, a change in expression of genes related to fatty acid metabolism in IVF porcine embryos during the maternal-to-zygotic transition (MZT) was reported, suggesting the important role of this pathway during early development of porcine embryos [29]. Genes related to gap junctions are also crucial during early development, being required for normal cell communication that controls cell growth and differentiation, ensuring the success of compaction and cavitation [30].

miR-100-5p is upregulated in serum-derived exosomes from women with viable intrauterine pregnancy versus women with spontaneous abortion and ectopic pregnancy [31]. The downregulation of miR-100 in whole peripheral blood is associated with pregnancy complications in women [32,33]. However, in this work, miR-100 was more represented within EVs secreted by non-competent embryos arrested during compaction. Using DIANA-Tarbase v8, a target mRNA for miR-100 was identified. This gene, SMARCA5 (SWI/SNF Related, Matrix Associated, Actin Dependent Regulator of Chromatin, Subfamily A, Member 5), also known as SNF2H (Human Sucrose Nonfermenting Protein 2 Homologue), is part of the ATP-dependent chromatin remodelers [34]. In mice embryos, the loss of SNF2H resulted in developmental arrest and death of the inner cell mass and trophectoderm cells [35]. Torres-Padilla and Zernick-Goetz also showed SNF2H as a crucial gene for mice embryo development, because the loss of its expression led to developmental arrest in early stages [36]. Furthermore, SNF2H is necessary for proliferation of early blastocyst-derived stem cells, and its absence prevented the development and differentiation of these cells [35].

Another miRNA, bta-miR-502, upregulated in EVs from non-competent embryos, targets different mRNAs that have contributions in tissue development and differentiation. For example, GLCCI1 (Glucocorticoid-induced transcript 1) is a gene necessary for glomerular development in mice and zebrafish embryos [37]. ATXN1 (Ataxin) has an important role in neurogenesis, and miR-502 can decrease its expression [38]; miR-502 also targets NRBF2 (Nuclear receptor binding factor 2), which is decreased in mouse embryos. Recently NRBF2 was implicated in altering functions related to the nervous system [39]. Regarding miR-103, one of its targets is DGCR8, and the knockout of this gene led to the early developmental arrest of mouse embryos [40]. Therefore, the high levels of miR-502, 103, and 100 in EVs secreted by arrested embryos (G1) could potentially be correlated with the low competence of these embryos.

Conversely, bta-miR-140, bta-miR-92a, bta-miR-222, and bta-miR-2285 are upregulated in EVs from competent embryos. If it is assumed that there is a positive relation between the level of miRNA in EVs and in the embryo, it could be assumed that these miRNAs are also overexpressed in the corresponding embryo. However, the downregulation of mir-140 in rat embryos is essential for implantation [41]. The administration of miR-140 mimic reduces the number of implantation sites in the rat uterus, downregulating the expression of IGF1R in endometrial epithelial cells [41]. In bovine embryos, IGF1R is crucial for growth and development prior to implantation [42], suggesting that miR-140 should be downregulated in embryos. Moreover, miR-140 downregulates SOX2 [43], whose expression starts at the 16-cell stage in bovine embryos and which has an important role maintaining the pluripotency program [44].

A high expression of miR-92a has been related with the downregulation of the expression of genes related to cell adhesion [45]. One of the morphological changes that occurs at the eight-cell stage is compaction, which involves a smoothening of the surface of the embryo, associated with an increase of intercellular adhesion mediated by E-cadherin [46]. The decrease of E-cadherin-mediated cell adhesion will lead to an incorrect segregation of trophectoderm and inner-cell-mass cell fate [47], inducing developmental arrest [48]. Moreover, miR-92a has ASCL2 as a target gene [49], which is required for the maintenance of glycogen in trophoblast cells during development. A low level of ASCL2 mRNA level leads to the failure of mice placenta development, and its absence leads to embryonic lethality [50].

The upregulation of miR-222 is related to an abnormal invasion of trophoblast, inducing apoptosis by downregulating BCL2-L11 expression via targeting the 3′ untranslated region of the gene [51]. The low level of proapoptotic BCL2 family members leads to apoptosis in bovine embryos [52]. Hence, the high expression of miR-222 will be detrimental for the embryo. However, considering the pattern of miR-140, miR-222, and miR-92 in EVs secreted by competent embryos and the function of these miRNAs, it seems that EVs might act as a discard mechanism of molecules that are harmful for embryo development. This theory has also been suggested in other reports; thus, the miRNA content in EVs does not necessarily reflect the content in the secreting cell [12,53].

Based on the revised literature and the results from this work, it seems that embryos secrete EVs with a miRNA content according to embryo competence and developmental stage. In bovine embryos, at the eight-cells stage, a replacement of maternal mRNA transcripts by new embryonic transcripts occurs [18]. This can be accompanied by an intentional discarding of miRNAs. During the studied developmental period, EVs secreted by arrested embryos had a bigger size than EVs secreted by embryos that reached blastocyst stage. It could be possible that EVs collected from bad-quality embryos are more heterogeneous, having the contribution of microvesicles and apoptotic bodies. Due to the origin of these vesicles, it could be expected that there is a correlation between EVs and blastomere content. However, in good-quality embryos, the exosome pathway could be favored. In this case, the miRNAs within EVs secreted by good embryos may correspond to discarded molecules. We concluded that the loading of specific miRNAs into the EVs during the compaction period depends on embryo competence.

## 4. Materials and Methods

All experiments were approved by the Ethics Committee of the Faculty of Veterinary Sciences, University of Concepcion, Chile (permit no. CBE-17-2017).

### 4.1. General Design

In this work, the characteristics of nanoparticles secreted by bovine embryos at the window between embryo genome activation (EGA) and embryo compaction were analyzed. For this, embryos were produced by in vitro fertilization and cultured in groups until day 3.5; at this point, only 8-to-16-cell embryos were selected and further cultured individually, until day 5, on EVs depleted culture media. Culture media were collected individually, at day 5, for the separation and characterization of nanoparticles, and then the embryos were kept in culture until day 7, for the final morphological evaluation. Culture media were then classified according to the capacity of their embryo to reach blastocyst stage at day 7: arrested embryos on 8-to-16-cell stage (G1) and embryos that reached blastocyst stage (G2). Separation of nanoparticles was carried out, using our standardized protocol [12]. Nanoparticles tracking analysis (NTA) was performed in individual culture media, to characterize the nanoparticles according to concentration and size. Transmission electronic microscopy (TEM) and flow cytometry were performed to determine the presence of extracellular vesicles secreted by embryos. After NTA, 3 pools of 10 culture media per group of individually cultured embryos were analyzed by next-generation sequencing (NGS). Finally, bioinformatics analysis was performed to obtain the micro-RNAs differentially expressed in G1 versus G2.

### 4.2. In Vitro Embryo Production

Bovine embryos were in vitro produced by following standard procedures and using culture medium described by Velasquez et al. [54]. The cumulus–oocyte complexes (COCs) were collected and in vitro matured (IVM) for 24 h in four-well dishes (25 to 30 COCs per 500 µL), using TCM-199 medium supplemented with follicle stimulating and luteinizing hormones (0.01 U/mL each), 17β-estradiol (1 μg/mL), epidermal growth factor (EGF; 10 ng/mL), and 10% FBS, at 39 °C, in a 5% CO_2_, in air atmosphere. COCs were in vitro fertilized (IVF = day 0) by using frozen-thawed commercial semen (ABS Global, Chile) previously tested in our laboratory. After 18–20 h of IVF, cumulus cells were mechanically removed from presumptive zygotes before in vitro culture (IVC). IVC was carried out in groups on 4-well dishes (25 to 30 COCs per 500 µL), in synthetic oviduct fluid (SOF) supplemented with 0.37 mM trisodium citrate, 2.77 mM myo-inositol, essential and non-essential amino acids (final concentration 1×, gentamycin (50 ug/mL), 3 mg/mL essentially fatty acid free BSA, 2% FBS, and 10 ng/mL EGF. At day 3.5, only the 8-to-16-cell embryos were selected for individual culture in EVs-depleted SOF (80 µL). EVs-depleted SOF (SOFd) was produced by ultrafiltration (centrifugal filter devices 100 kDa, Amicon) of complete SOF, for 15 min, at 1660× *g*, at 4 °C. Each conditioned-culture medium was collected at day 5, conserved individually at −80 °C and identified with the corresponding embryo. The embryos continued in culture on fresh SOF until day 7, for morphological evaluation, according IETS criteria [55]. Each conditioned-culture medium was classified according to the capacity of its embryo to reach blastocyst stage at day 7 (G1 and G2) and kept for EVs analyses.

### 4.3. Separation of EVs from Conditioned-Culture Media by Early Embryos

To characterize the molecular and morphological characteristics of nanoparticles secreted by preimplantation bovine embryos from day 3.5 to day 5, independent samples were generated. The culture medium from 100 embryos cultured individually were pooled for each group (G1 and G2) and the nanoparticles’ separation was carried out by using the method described by Théry et al. [56], with modifications. Sterile PBS was added to the pool of 100 culture media, to complete 15 mL. Several steps of centrifugation were performed to eliminate cells and cell debris (700× *g* for 10 min; 2000× *g* for 10 min; 10,000× *g* for 70 min). The last supernatant was centrifuged overnight at 100,000× *g*, and the pellet was resuspended in 0.5 mL of PBS. Nanoparticle concentration was carried out by ultrafiltration, using centrifugal filter devices (0.5 mL, 10 kDa, Amicon, Merck, Darmstadt, Germany), for 20 min, at 2500× *g*; 20 μL was kept frozen, at −20 °C, for further analyses by TEM and flow cytometry analyses.

To determine the concentration and size distribution of EVs and their content, new batches of samples were produced. Culture media were collected individually, identified with their corresponding embryo, and then classified as G1 or G2 according to embryo development. Five hundred microliters of sterile PBS were added to each sample (60 μL). Then, two successive centrifugations (700× *g* for 10 min; 2000× *g* for 10 min) were carried out. Subsequently, the recovered supernatants were analyzed according Mellisho et al. [11]. NTA was performed to determine concentration and size distribution of EVs. Each sample was individually recovered after NTA and then polled to organize three replicates of 10 either for G1 and G2, for small RNA-sequencing. EVs concentration was carried out by ultrafiltration, using centrifugal filter devices (0.5 mL, 10 kDa, Amicon, Merck, Darmstadt, Germany), for 20 min, at 2500× *g*.

### 4.4. Morphological and Molecular Characterization of Nanoparticles from Conditioned-Culture Media by Early Embryos

#### 4.4.1. Flow Cytometer for EVs Markers

The phenotype of EVs was evaluated by identification of surface markers, using flow cytometry, following the protocol described by Théry et al. [56], with modifications. EVs (4 × 10^8^ particles/mL) were incubated with 4 μm aldehyde/sulfate latex beads (1.25 × 10^5^ particles/mL; ThermoFisher Scientific, Waltham, MA, USA). The EVs/beads complexes were incubated with primary antibodies against CD63 (FITC-conjugated; catalog no. 18235, Abcam, Cambridge, UK), CD9 (FITC-conjugated; catalog no. 34162, Abcam, Cambridge, UK), CD81 (PE-conjugated; catalog no. 81436, Abcam, Cambridge, UK), and CD40L (PE/Cy5^®^-conjugated; catalog no. 25044, Abcam, Cambridge, UK), for 2 h, at 4 °C. EVs isolated from bovine follicular fluid and human cells culture supernatant were used as positive controls [42]. A negative control antibody reaction was performed by using latex beads, alone, incubated with each antibody, for 2 h, at 4 °C. The labeled EVs/beads complexes were resuspended in focusing fluid and subjected to flow cytometry, using Attune™ NxT Flow Cytometer (ThermoFisher Scientific, Waltham, MA, USA).

#### 4.4.2. Transmission Electron Microscopy (TEM) Analysis

TEM was used to identify the morphology of EVs from G1 and G2. The separated nanoparticles were deposited on formvar-carbon-coated copper grids for whole-mount preparations and subjected to TEM analysis, using the protocol described by Théry et al. [56], with slight modifications. Briefly, 10 μL of collected nanoparticles was thawed and mixed with an equal volume of 4% paraformaldehyde. Two grids were prepared for each group, washed, and fixed with 1% glutaraldehyde and then contrasted with uranyl-oxalate solution (pH 7.0) and 4% uranyl-acetate.

Grids were observed in “Centro de Espectroscopía y Microscopía” (CESMI, Universidad de Concepción, Concepción, Chile) on a JEOL JEM 1200 EXII transmission electron microscope, provided with a Gatan 782 camera. The equipment had a resolution of 5A and was operated at 80 kV. The images were processed by using the software provided from the equipment.

#### 4.4.3. Nanoparticles Tracking Analysis (NTA)

After two successive centrifugations (described above), the recovered supernatants from individual culture media were subjected to NTA, to determine the presence, concentration, and size distribution of nanoparticles. For this, a NanoSight NS300 (Malvern Instruments Ltd., Malvern, UK) equipped with a 488 nm and sCMOS camera was used. EVs-depleted media used for embryo culture were also analyzed as negative control. EVs characteristics were determined at 20 to 100 particles per frame. Negative control had less than seven particles per frame. Samples were injected in a continuous flow into the sample chamber, at room temperature (RT), using a syringe pump. Analysis of each sample was performed as described by Mellisho et al. [11] and considering each experimental group. Graphical analysis showed size distribution of the nanoparticles per experimental group, and the concentration was reported as particles per milliliter.

### 4.5. miRNAs Cargo Characterization of EVs from Conditioned-Culture Media by Early Embryos

After completing NTA, the individual culture media were recovered and pooled, to organize three biological replicates of 10 for G1 and G2, for small RNA sequencing. EVs were concentrated by using ultrafiltration with Amicon filter devices, as described above.

#### 4.5.1. Extracellular Vesicles RNA Isolation and Quantification

All the samples were submitted to Norgen facilities for Next-Generation Sequencing (NGS). The whole RNA was isolated by using Plasma/Serum RNA Purification Mini Kit (Norgen Biotek, Thorold, ON, Canada). Ribogreen kit assay (Thermofisher, Waltham, MA, USA) was performed for the quantification in a microplate reader, at 260 nm, using 1 μL of RNA, and the quality was measured with the Agilent RNA Pico chip kit (Agilent Technologies, Santa Clara, CA, USA).

#### 4.5.2. Small RNAs Sequencing Analysis

Before RNA sequencing, the libraries were constructed, using Small RNA Library Prep Kit and following manufacturer’s instructions (Norgen Biotek, Thorold, Canada). Then, small RNA sequencing was performed in Illumina NextSeq 500 platform with the NextSeq 500/550 High Output kit v2 (Illumina, San Diego, CA, USA). The quality of resulting libraries was analyzed by using FastQC software (Babraham Bioinformatics, Cambridge, UK). Adaptor sequences were trimmed, aligned, and counted, using SRNA bench pipeline [57]. For the miRNA library, we accepted reads with values above 30 Phreads and 18 to 30 bp of length, resulting in 24,336,044 reads for mapping. The reads were mapped against the reference genome ARS-UCD 1.2 and miRbase database, using Bowtie2 software and miRdeep2 mapper. Gene counts were calculated by using HTSeq, based on a filtration of counts per million (CPM) higher or equivalent to 5.

### 4.6. Statistical Analysis

Measurable variables of EVs (mean size and concentration) were analyzed with a nonparametric Wilcoxon test. Differences were considered significant with a *p*-value < 0.05. Analysis was carried out with the statistical program InfoStat (Buenos Aires, Argentina, 2002).

Differential miRNA expression analysis was performed by using the resulting list of miRNAs and carried out by using EdgeR package. Two criteria were applied to discriminate upregulated and downregulated miRNAs: *p*-value < 0.05 and logarithm of the fold change of <−0.5 and >0.5. The results were plotted in Volcano Plot, using ggplot2 package. Finally, the identified set of differentially expressed miRNAs was used for pathway enrichment analysis, using an online tool, mirPath 3.0 [58], based on Kyoto Encyclopedia of Genes and Genome (KEGG) pathways. Prediction was based on Tarbase algorithms. The statistical significance value associated with the biological pathway was calculated by mirPath software.

## Figures and Tables

**Table 1:** MiRNAs identified in extracellular vesicles secreted by bovine embryos during the compaction period.

miR	Significance Comparing G1 vs. G2	Gene Expression Respect to G2
bta-miR-103	0.0003	Increased
bta-miR-100	0.0004	Increased
bta-miR-502a	0.0097	Increased
bta-miR-1	0.0208	Increased
bta-miR-101	0.1199	Increased
bta-let-7e	0.1682	Increased
bta-miR-215	0.1876	Increased
bta-miR-205	0.2915	Increased
bta-miR-193a-3p	0.4369	Increased
bta-miR-224	0.4895	Increased
bta-miR-222	0.0034	Decreased
bta-miR-92a	0.0323	Decreased
bta-miR-2285av	0.0385	Decreased
bta-miR-140	0.0429	Decreased
bta-miR-25	0.0545	Decreased
bta-miR-9-5p	0.0692	Decreased
bta-miR-16b	0.0824	Decreased
bta-miR-191	0.1003	Decreased
bta-miR-744	0.1038	Decreased
bta-miR-451	0.1163	Decreased
bta-miR-10b	0.1211	Decreased
bta-let-7f	0.1519	Decreased
bta-miR-2904	0.1561	Decreased
bta-miR-11980	0.1609	Decreased
bta-let-7g	0.1609	Decreased
bta-miR-30d	0.1714	Decreased
bta-miR-93	0.1873	Decreased
bta-miR-378	0.1994	Decreased
bta-let-7i	0.2239	Decreased
bta-miR-148b	0.2253	Decreased
bta-miR-143	0.2362	Decreased
bta-miR-144	0.2412	Decreased
bta-miR-151-3p	0.2510	Decreased
bta-miR-21-5p	0.2891	Decreased
bta-miR-221	0.3007	Decreased
bta-miR-152	0.3234	Decreased
bta-miR-455-5p	0.3246	Decreased
bta-miR-199a-3p	0.3564	Decreased
bta-miR-11995	0.3646	Decreased
bta-miR-200a	0.3960	Decreased
bta-miR-96	0.4180	Decreased
bta-miR-124b	0.4465	Decreased
bta-miR-124a	0.4622	Decreased
bta-miR-15b	0.4951	Decreased
bta-miR-92b	0.5279	Decreased
bta-miR-486	0.1387	Equal
bta-miR-10a	0.2450	Equal
bta-miR-423-5p	0.3259	Equal
bta-let-7c	0.3432	Equal
bta-miR-27a-3p	0.3529	Equal
bta-miR-1246	0.3565	Equal
bta-miR-27b	0.3948	Equal
bta-let-7b	0.4056	Equal
bta-miR-183	0.4368	Equal
bta-miR-151-5p	0.5007	Equal
bta-miR-142-5p	0.5462	Equal
bta-miR-302b	0.5876	Equal
bta-miR-24-3p	0.5886	Equal
bta-miR-181a	0.5970	Equal
bta-miR-29b	0.5970	Equal
bta-miR-99a-5p	0.6146	Equal
bta-miR-29d-3p	0.6151	Equal
bta-miR-29c	0.6178	Equal
bta-miR-320a	0.6476	Equal
bta-miR-125b	0.6481	Equal
bta-miR-192	0.6536	Equal
bta-miR-29a	0.6548	Equal
bta-miR-125a	0.6586	Equal
bta-miR-484	0.6634	Equal
bta-miR-26b	0.6787	Equal
bta-miR-199a-5p	0.6898	Equal
bta-miR-30a-5p	0.7072	Equal
bta-miR-22-3p	0.7340	Equal
bta-miR-146a	0.7652	Equal
bta-miR-375	0.7673	Equal
bta-miR-2478	0.7729	Equal
bta-miR-122	0.7755	Equal
bta-miR-141	0.7853	Equal
bta-miR-30e-5p	0.7925	Equal
bta-miR-146b	0.7971	Equal
bta-miR-26a	0.8062	Equal
bta-miR-148a	0.8207	Equal
bta-miR-99b	0.8406	Equal
bta-miR-184	0.8484	Equal
bta-miR-7	0.8697	Equal
bta-miR-23b-3p	0.8705	Equal
bta-miR-423-3p	0.8750	Equal
bta-miR-30c	0.8867	Equal
bta-miR-10174-3p	0.8955	Equal
bta-let-7a-5p	0.9635	Equal
bta-miR-182	0.9746	Equal
bta-miR-23a	0.9817	Equal
bta-miR-186	0.9912	Equal
bta-miR-185	1	Equal
bta-miR-2473	1	Equal

Significance: *p* < 0.05. Differential expression was performed between G1 and G2. G1: conditioned-culture media by embryos arrested in the 8–16-cells stage. G2: conditioned-culture media by embryos that reached blastocyst stages at day 7.

**Table 2:** Detected miRNA number for differential expression analysis.

KEEG Pathway	*p*-Value	Target Genes	miRNA
Fatty acid biosynthesis	0.000005	1	1
Fatty acid metabolism	0.00017	4	2
Lysine degradation	0.00089	9	4
Gap junction	0.00089	19	4
Signaling pathways regulating pluripotency of stem cells	0.0046	27	4

## Abbreviations

EVs	Extracellular vesicles
miR	microRNA
TEM	Transmission electron microscopy
NTA	Nanoparticles tracking analysis
NGS	Next-generation sequencing
G1	Group 1: embryos arrested on 8–16-cells stage at day 3.5 of culture
G2	Group 2: embryos that reached blastocyst stage at day 7 of culture
IVF	In vitro fertilization
EGA	Embryo genome activation
SOF	Synthetic oviduct fluid
SOFd	EVs-depleted SOF

## References

[1] Cocucci E., Racchetti G., Meldolesi J. (2009). Shedding microvesicles: Artefacts no more. Trends Cell Biol..

[2] György B., Szabó T.G., Pásztói M., Pál Z., Misják P., Aradi B., László V., Pállinger É., Pap E., Kittel Á. (2011). Membrane vesicles, current state-of-the-art: Emerging role of extracellular vesicles. Cell. Mol. Life Sci..

[3] Yáñez-Mó M., Siljander P., Andreu Z., Zavec A.B., Borràs F.E., Buzas E.I., Buzas K., Casal E., Cappello F., Carvalho J. (2015). Biological properties of extracellular vesicles and their physiological functions. J. Extracell. Vesicles.

[4] Hill A.F., Pegtel D.M., Lambertz U., Leonardi T., O’Driscoll L., Pluchino S., Ter-Ovanesyan D., Nolte-‘t Hoen E.N. (2013). ISEV position paper: Extracellular vesicle RNA analysis and bioinformatics. J. Extracell. Vesicles.

[5] Raposo G., Stoorvogel W. (2013). Extracellular vesicles: Exosomes, microvesicles, and friends. J. Cell Biol..

[6] Machtinger R., Laurent L.C., Baccarelli A.A. (2016). Extracellular vesicles: Roles in gamete maturation, fertilization and embryo implantation. Hum. Reprod. Update.

[7] Burns G., Brooks K., Wildung M., Navakanitworakul R., Christenson L.K., Spencer T.E. (2014). Extracellular Vesicles in Luminal Fluid of the Ovine Uterus. PLoS ONE.

[8] Saadeldin I.M., Kim S.J., Bin Choi Y., Lee B.C. (2014). Improvement of Cloned Embryos Development by Co-Culturing with Parthenotes: A Possible Role of Exosomes/Microvesicles for Embryos Paracrine Communication. Cell. Reprogram..

[9] Giacomini E., Vago R., Sanchez A.M., Podini P., Zarovni N., Murdica V., Rizzo R., Bortolotti D., Candiani M., Viganò P. (2017). Secretome of in vitro cultured human embryos contains extracellular vesicles that are uptaken by the maternal side. Sci. Rep..

[10] Pavani K.C., Hendrix A., Broeck W.V.D., Couck L., Szymanska K., Lin X., De Koster J., Van Soom A., Leemans B. (2018). Isolation and Characterization of Functionally Active Extracellular Vesicles from Culture Medium Conditioned by Bovine Embryos In Vitro. Int. J. Mol. Sci..

[11] Mellisho E., Velasquez A.E., Núñez M.J., Cabezas J.G., Cueto J.A., Fader C., Castro F.O., Rodriguez-Alvarez L. (2017). Identification and characteristics of extracellular vesicles from bovine blastocysts produced in vitro. PLoS ONE.

[12] Mellisho E.A., Briones M.A., Velasquez A.E., Cabezas J., Castro F.O., Rodriguez-Alvarez L. (2019). Extracellular vesicles secreted during blastulation show viability of bovine embryos. Reproduction.

[13] Dissanayake K., Nõmm M., Lättekivi F., Ressaissi Y., Godakumara K., Lavrits A., Midekessa G., Viil J., Bæk R., Jørgensen M.M. (2020). Individually cultured bovine embryos produce extracellular vesicles that have the potential to be used as non-invasive embryo quality markers. Theriogenology.

[14] Cha J., Sun X., Dey S.K. (2012). Mechanisms of implantation: Strategies for successful pregnancy. Nat. Med..

[15] Song Y., An X., Zhang L., Fu M., Peng J., Han P., Hou J., Zhou Z., Cao B. (2015). Identification and Profiling of microRNAs in Goat Endometrium during Embryo Implantation. PLoS ONE.

[16] Ozawa M., Sakatani M., Yao J., Shanker S., Yu F., Yamashita R., Wakabayashi S., Nakai K., Dobbs K.B., Sudano M.J. (2012). Global gene expression of the inner cell mass and trophectoderm of the bovine blastocyst. BMC Dev. Biol..

[17] Rizos D., Clemente M., Bermejo-Alvarez P., De La Fuente J., Lonergan P., Gutiérrez-Adán A. (2008). Consequences ofIn VitroCulture Conditions on Embryo Development and Quality. Reprod. Domest. Anim..

[18] Sirard M.-A. (2010). Activation of the embryonic genome. Reprod. Domest. Rumin..

[19] Mondou E., Dufort I., Gohin M., Fournier E., Sirard M.-A. (2012). Analysis of microRNAs and their precursors in bovine early embryonic development. Mol. Hum. Reprod..

[20] Tripurani S.K., Wee G., Lee K.-B., Smith G.W., Wang L., Yao J. (2013). MicroRNA-212 Post-Transcriptionally Regulates Oocyte-Specific Basic-Helix-Loop-Helix Transcription Factor, Factor in the Germline Alpha (FIGLA), during Bovine Early Embryogenesis. PLoS ONE.

[21] Théry C., Witwer K.W., Aikawa E., Alcaraz M.J., Anderson J.D., Andriantsitohaina R., Antoniou A., Arab T., Archer F., Atkin-Smith G.K. (2018). Minimal information for studies of extracellular vesicles 2018 (MISEV2018): A position statement of the International Society for Extracellular Vesicles and update of the MISEV2014 guidelines. J. Extracell. Vesicles.

[22] Ferreira Y.J., Gardiner C., Poli M., Turner K., Child T., Sargent I.L. (2013). O-253 Human embryos release extracellular vesicles which may act as indicators of embryo quality. Session 66: Embryo quality: Does it predict pregnancy?. Hum. Reprod..

[23] Abu-Halima M., Häusler S., Backes C., Fehlmann T., Staib C., Nestel S., Nazarenko I., Meese E., Keller A. (2017). Micro-ribonucleic acids and extracellular vesicles repertoire in the spent culture media is altered in women undergoing In Vitro Fertilization. Sci. Rep..

[24] Pallinger E., Bognar Z., Bodis J., Csabai T., Farkas N., Godony K., Varnagy A., Buzas E., Szekeres-Bartho J. (2017). A simple and rapid flow cytometry-based assay to identify a competent embryo prior to embryo transfer. Sci. Rep..

[25] Almiñana C., Tsikis G., Labas V., Uzbekov R., Da Silveira J.C., Bauersachs S., Mermillod P. (2018). Deciphering the oviductal extracellular vesicles content across the estrous cycle: Implications for the gametes-oviduct interactions and the environment of the potential embryo. BMC Genom..

[26] Ng Y.H., Rome S., Jalabert A., Forterre A., Singh H., Hincks C.L., Salamonsen L.A. (2013). Endometrial Exosomes/Microvesicles in the Uterine Microenvironment: A New Paradigm for Embryo-Endometrial Cross Talk at Implantation. PLoS ONE.

[27] Zhang Y., Zhou J., Li M.-Q., Xu J., Zhang J.-P., Jin L.-P. (2019). MicroRNA-184 promotes apoptosis of trophoblast cells via targeting WIG1 and induces early spontaneous abortion. Cell Death Dis..

[28] Lin X., Pavani K.C., Smits K., Deforce D., Heindryckx B., Van Soom A., Peelman L. (2019). Bta-miR-10b Secreted by Bovine Embryos Negatively Impacts Preimplantation Embryo Quality. Front. Genet..

[29] Xiang J., Xing Y., Long C., Hou D., Liu F., Zhang Y., Lu Z., Wang J., Zuo Y., Li X. (2020). Fatty acid metabolism as an indicator for the maternal–to–zygotic transition in porcine IVF embryos revealed by RNA sequencing. Theriogenology.

[30] Sugimoto M., Sasaki S., Gotoh Y., Nakamura Y., Aoyagi Y., Kawahara T., Sugimoto Y. (2013). Genetic variants related to gap junctions and hormone secretion influence conception rates in cows. Proc. Natl. Acad. Sci. USA.

[31] Sun J., Deng G., Ruan X., Chen S., Liao H., Liu X., Li J., Zhao G., Gaopi D. (2020). Exosomal MicroRNAs in Serum as Potential Biomarkers for Ectopic Pregnancy. BioMed. Res. Int..

[32] Hromadnikova I., Kotlabova K., Hympanova L., Krofta L. (2016). Gestational hypertension, preeclampsia and intrauterine growth restriction induce dysregulation of cardiovascular and cerebrovascular disease associated microRNAs in maternal whole peripheral blood. Thromb. Res..

[33] Yang Q., Gu W.-W., Gu Y., Yan N.-N., Mao Y.-Y., Zhen X.-X., Wang J.-M., Yang J., Shi H.-J., Zhang X. (2018). Association of the peripheral blood levels of circulating microRNAs with both recurrent miscarriage and the outcomes of embryo transfer in an in vitro fertilization process. J. Transl. Med..

[34] Wilson B.G., Roberts C.W.M. (2011). SWI/SNF nucleosome remodellers and cancer. Nat. Rev. Cancer.

[35] Stopka T., Skoultchi A.I. (2003). The ISWI ATPase Snf2h is required for early mouse development. Proc. Natl. Acad. Sci. USA.

[36] Torres-Padilla M.E., Zernicka-Goetz M. (2006). Role of TIF1α as a modulator of embryonic transcription in the mouse zygote. J. Cell Biol..

[37] Nishibori Y., Katayama K., Parikka M., Oddsson A., Nukui M., Hultenby K., Wernerson A., He B., Ebarasi L., Raschperger E. (2011). Glcci1 Deficiency Leads to Proteinuria. J. Am. Soc. Nephrol..

[38] Lee Y., Fryer J.D., Kang H., Crespo-Barreto J., Bowman A.B., Gao Y., Kahle J.J., Hong J.S., Kheradmand F., Orr H.T. (2011). ATXN1 Protein Family and CIC Regulate Extracellular Matrix Remodeling and Lung Alveolarization. Dev. Cell.

[39] Ouyang X., Ahmad I., Johnson M.S., Redmann M., Craver J., Wani W.Y., Benavides G.A., Chacko B., Li P., Young M. (2020). Nuclear receptor binding factor 2 (NRBF2) is required for learning and memory. Lab. Investig..

[40] Wang Y., Medvid R., Melton C., Jaenisch R., Blelloch R. (2007). DGCR8 is essential for microRNA biogenesis and silencing of embryonic stem cell self-renewal. Nat. Genet..

[41] Sirohi V.K., Gupta K., Kumar R., Shukla V., Dwivedi A. (2018). Selective estrogen receptor modulator ormeloxifene suppresses embryo implantation via inducing miR-140 and targeting insulin-like growth factor 1 receptor in rat uterus. J. Steroid Biochem. Mol. Biol..

[42] McCarthy S.D., Roche J.F., Forde N. (2012). Temporal changes in endometrial gene expression and protein localization of members of the IGF family in cattle: Effects of progesterone and pregnancy. Physiol. Genom..

[43] Green D., Dalmay T., Fraser W.D. (2015). Role of miR-140 in embryonic bone development and cancer. Clin. Sci..

[44] Goissis M.D., Cibelli J.B. (2014). Functional characterization of CDX2 during bovine preimplantation development in vitro. Mol. Reprod. Dev..

[45] Yamada N., Heishima K., Akao Y., Senda T. (2019). Extracellular Vesicles Containing MicroRNA-92a-3p Facilitate Partial Endothelial-Mesenchymal Transition and Angiogenesis in Endothelial Cells. Int. J. Mol. Sci..

[46] Shirayoshi Y., Okada T.S., Takeichi M. (1983). The calcium-dependent cell-cell adhesion system regulates inner cell mass formation and cell surface polalrization in early mouse development. Cell.

[47] Stephenson R.O., Yamanaka Y., Rossant J. (2010). Disorganized epithelial polarity and excess trophectoderm cell fate in preimplantation embryos lacking E-cadherin. Development.

[48] Sathanawongs A., Nganvongpanit K., Mekchay S. (2012). Expression patterns of cell adhesion molecules in bovine preimplantation embryos cultured in vitro. Thai J. Vet. Med..

[49] Karagkouni D., Paraskevopoulou M.D., Chatzopoulos S., Vlachos I.S., Tastsoglou S., Kanellos I., Papadimitriou D., Kavakiotis I., Maniou S., Skoufos G. (2017). DIANA-TarBase v8: A decade-long collection of experimentally supported miRNA–gene interactions. Nucleic Acids Res..

[50] Bogutz A.B., Oh-McGinnis R., Jacob K.J., Ho-Lau R., Gu T., Gertsenstein M., Nagy A., Lefebvre L. (2018). Transcription factor ASCL2 is required for development of the glycogen trophoblast cell lineage. PLoS Genet..

[51] Qu H.-M., Qu L.-P., Pan X.-Z., Mu L.-S. (2018). Upregulated miR-222 targets BCL2L11 and promotes apoptosis of mesenchymal stem cells in preeclampsia patients in response to severe hypoxia. Int. J. Clin. Exp. Pathol..

[52] Fear J.M., Hansen P.J. (2011). Developmental Changes in Expression of Genes Involved in Regulation of Apoptosis in the Bovine Preimplantation Embryo. Biol. Reprod..

[53] Maida Y., Takakura M., Nishiuchi T., Yoshimoto T., Kyo S. (2016). Exosomal transfer of functional small RNAs mediates cancer-stroma communication in human endometrium. Cancer Med..

[54] Velasquez A.E., Castro F.O., Veraguas D., Cox J.F., Lara E., Briones M., Rodriguez-Alvarez L. (2014). Splitting of IVP bovine blastocyst affects morphology and gene expression of resulting demi-embryos during in vitro culture and in vivo elongation. Zygote.

[55] Bó G.A., Mapletoft R.J. (2013). Evaluation and classification of bovine embryos. Anim. Reprod..

[56] Théry C., Amigorena S., Raposo G., Clayton A. (2006). Isolation and Characterization of Exosomes from Cell Culture Supernatants and Biological Fluids. Curr. Protoc. Cell Biol..

[57] Barturen G., Rueda A., Hamberg M., Alganza A., Lebron R., Kotsyfakis M., Shi B.-J., Koppers-Lalic D., Hackenberg M. (2014). sRNAbench: Profiling of small RNAs and its sequence variants in single or multi-species high-throughput experiments. Methods Next Gener. Seq..

[58] Vlachos I.S., Zagganas K., Paraskevopoulou M.D., Georgakilas G., Karagkouni D., Vergoulis T., Dalamagas T., Hatzigeorgiou A.G. (2015). DIANA-miRPath v3.0: Deciphering microRNA function with experimental support. Nucleic Acids Res..

